# Supplementary material for: Causal Relationship between Aging and Anorexia Nervosa: A White-Matter-Microstructure-Mediated Mendelian Randomization Analysis
Source: Biomedicines. 2024 Aug 16;12(8):1874. doi: 10.3390/biomedicines12081874 (PMC11351342; doi:10.3390/biomedicines12081874)
Supplement: Supplementary file 1 [file biomedicines-12-01874-s001.zip › Supplementary Figure/Figure S8/Leaveoneout plot of MR analysis for TL to ubm-b-1814.pdf]

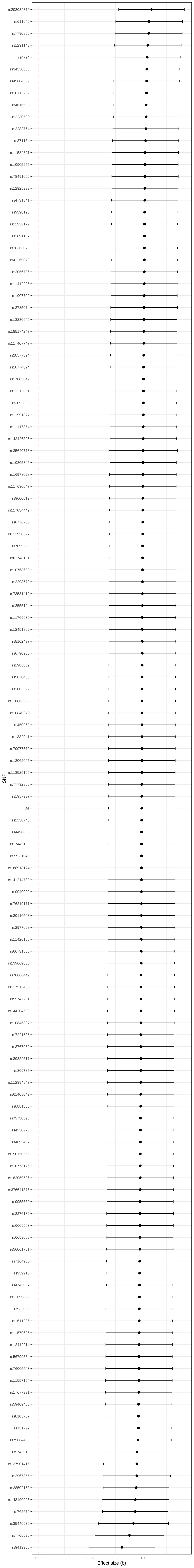
rs202034370
rs611646
rs7790856
rs1291143
rs4724
rs34550383
rs45604339
rs10112752
rs4616688
rs2230590
rs2282764
rs871134
rs11584821
rs10905255
rs78491606
rs12925933
rs4731541
rs9398196
rs12932179
rs3891167
rs28363070
rs41269079
rs2056726
rs11412296
rs1907702
rs3785074
rs13230646
rs185174247
rs117407747
rs28577594
rs10774624
rs17803849
rs11212631
rs3093888
rs11991877
rs11117354
rs142426306
rs35640778
rs10805346
rs16978028
rs117630647
rs9600019
rs117034449
rs6776756
rs111950327
rs7099229
rs61748181
rs10768683
rs2293579
rs73581419
rs2555104
rs11769630
rs12451892
rs8102497
rs6790988
rs1985369
rs9878436
rs1003322
rs116863223
rs10840270
rs450962
rs1332941
rs79977579
rs13062095
rs113525195
rs77732866
rs1957937
All
rs2538745
rs4498805
rs17445108
rs77231040
rs188918174
rs141214782
rs9940099
rs76219171
rs80116508
rs2977608
rs11426156
rs66731853
rs139669835
rs76666449
rs117512405
rs55747751
rs144204502
rs10845387
rs7221585
rs3767952
rs80324517
rs869785
rs112394943
rs61405042
rs6881568
rs73730598
rs4530278
rs4695407
rs150150565
rs10773176
rs182059586
rs376641875
rs9955360
rs2276182
rs6669563
rs6659669
rs56061761
rs7164950
rs939916
rs4743037
rs11699829
rs932002
rs1611236
rs11579626
rs12412214
rs56799554
rs76065543
rs11557154
rs17677991
rs59409453
rs8105767
rs131797
rs75664430
rs5742915
rs137901416
rs2967355
rs28502153
rs143190905
rs762679
rs35446936
rs7705526
rs9419958
SNP
0.00
0.05
0.10
Effect size (b)
